# Transcriptional signature of lymphoblastoid cell lines of *BRCA1*, *BRCA2* and non-*BRCA1/2* high risk breast cancer families

**DOI:** 10.18632/oncotarget.20219

**Published:** 2017-08-12

**Authors:** Marie-Christine Pouliot, Charu Kothari, Charles Joly-Beauparlant, Yvan Labrie, Geneviève Ouellette, Jacques Simard, Arnaud Droit, Francine Durocher

**Affiliations:** ^1^ CHU de Québec Research Centre-Université Laval, Department of Molecular Medicine, Québec, Canada

**Keywords:** hereditary breast cancer, high-risk *BRCA1/2*/X families, gene expression, RNA-seq, lymphoblastoid cell lines

## Abstract

Approximately 25% of hereditary breast cancer cases are associated with a strong familial history which can be explained by mutations in *BRCA1* or *BRCA2* and other lower penetrance genes. The remaining high-risk families could be classified as BRCAX (non-*BRCA1/2*) families.

Gene expression involving alternative splicing represents a well-known mechanism regulating the expression of multiple transcripts, which could be involved in cancer development. Thus using RNA-seq methodology, the analysis of transcriptome was undertaken to potentially reveal transcripts implicated in breast cancer susceptibility and development.

RNA was extracted from immortalized lymphoblastoid cell lines of 117 women (affected and unaffected) coming from *BRCA1*, *BRCA2* and BRCAX families. Anova analysis revealed a total of 95 transcripts corresponding to 85 different genes differentially expressed (Bonferroni corrected p-value <0.01) between those groups. Hierarchical clustering allowed distinctive subgrouping of *BRCA1/2* subgroups from BRCAX individuals. We found 67 transcripts, which could discriminate BRCAX from *BRCA1/BRCA2* individuals while 28 transcripts discriminate affected from unaffected BRCAX individuals.

To our knowledge, this represents the first study identifying transcripts differentially expressed in lymphoblastoid cell lines from major classes of mutation-related breast cancer subgroups, namely *BRCA1*, *BRCA2* and BRCAX. Moreover, some transcripts could discriminate affected from unaffected BRCAX individuals, which could represent potential therapeutic targets for breast cancer treatment.

## INTRODUCTION

In 2015, breast cancer represented 26% of all cancer cases among Canadian women and was the second leading cause of cancer death constituting 14% of overall death due to cancer [1]. Like every common cancer, breast cancer shows some degree of familial clustering [2]. High-risk families having multiple cases of breast or ovarian cancer are associated with a higher risk of developing breast cancer during their lifetime than other families [3]. It is thought that approximately 10-15% of breast cancer cases are hereditary and associated with mutations in *BRCA1* or *BRCA2* genes and some other genes having high to moderate penetrance such as *TP53*, *PTEN*, *ATM*, *CHEK2*, *PALB2* and *BRIP1* and *ATR*, which account for approximately 5% of the risk [4-10]. Common variants have also been identified in additional susceptibility loci and would explain a further ∼16% of the 2-fold familial risk of breast cancer [11]. Among our French Canadian cohort, 24% of high-risk breast cancer families were found to be carriers of a deleterious *BRCA1* or *BRCA2* mutation [12].

Therefore, susceptibility alleles for more than half of the high-risk families remain unknown. A portion of these remnant breast cancer families could be explained by modulation of gene expression, which is mainly regulated through methylation or alternative splicing (AS) mechanisms. Indeed, more than 90% of human genes undergo alternative splicing and it is now becoming clear that AS plays an important role in human cancer development [13-14]. RNA sequencing allows a genome-wide expression study of the transcriptome and can likely detect and quantify all coding and non-coding transcripts [15]. The use of RNA sequencing greatly enhanced our understanding of gene expression in cells [16].

Human immortalized lymphoblastoid cell lines (LCLs) provide information on gene expression without having to consider tissue specific expression [17]. LCLs used for the establishment of gene expression or splicing signatures are recognized as a reliable biological material to study a given disease [18-28], and some studies recently showed the heritability of splicing, as some exons were spliced in an allele-specific manner [18, 29, 30]. Moreover, it has been demonstrated that LCLs can be used to study life-course environmental epigenetics [31].

Previous studies attempted to discriminate *BRCA1/2*, non-*BRCA1/2* (BRCAX) and sporadic breast cancers were based on gene expression levels and histological tests performed on breast tumor tissue [32-37]. In another study, although several genes or spliced transcripts were identified as differentially expressed in familial cases, they did not allow clusterization of *BRCA1*, *BRCA2* and BRCAX tumor tissues [38].

In this study, we performed RNA sequencing on LCLs isolated from *BRCA1/2* and BRCAX affected and unaffected individuals coming from high-risk breast cancer families in an attempt to distinguish breast cancer subgroups based on their transcriptome profile. This study revealed several transcripts involved in regulation of translation, apoptosis, cell cycle as well as cell growth and proliferation, which could discriminate BRCAX individuals from *BRCA1/2* subgroups.

## RESULTS

Our French Canadian cohort comprised three major familial breast cancer subgroups namely *BRCA1* and *BRCA2* carriers as well as BRCAX individuals, i.e. non *BRCA1/2* (affected and unaffected). The *BRCA1* cases included 25 individuals (ind) affected with breast cancer and 11 unaffected women, who were carriers of *BRCA1* mutations namely R1443X (22 ind), 3705insA (2 ind), 2244insA (7 ind), 2953del3+C (2 ind) and three individuals carrying E352X, 4160delAG or 1723del9ins13 mutation, respectively. The *BRCA2* subgroup was composed of 31 affected and 18 unaffected individuals carrying 8765delAG (44 ind), E3002K (2 ind), 6503delTT (1 ind), R3128X (1 ind) or 3036del4 mutation (1 ind). The BRCAX subgroup included 16 affected and 16 unaffected individuals, which represented 16 pairs of sisters (1 affected and 1 unaffected per family). It should be noted that the oldest unaffected sister available was purposely selected in BRCAX families. This subgroup of unaffected sisters was used as controls for comparison purpose in the analyses described below. The mutational profile and relationship status of *BRCA1, BRCA2* and *BRCAX* individuals are highlighted in Supplementary Table 1.

RNA-Seq analyses generated an average of 68 million reads per sample, and more than 85% of the reads were aligned to the hg19 human reference genome using TopHat (data not shown). As displayed in Supplementary Table 2, out of a total of 173 259 transcripts detected, 95 transcripts (0.05 % of all transcripts) were found to be significantly (p<0.01) and differentially expressed based on the Bonferroni-corrected ANOVA analysis, when considering all four breast cancer subgroups (*BRCA1*, *BRCA2*, unaffected and affected BRCAX individuals). All these significant transcripts were encoded by 85 different genes. In addition to the main isoforms (one per gene), 10 mRNA isoforms were considered as alternatively spliced isoforms (11.8%). These significant transcripts included 54 gene isoforms showing a highly significant Bonferroni-corrected p-value (p < 0.001).

**Figure 1:**
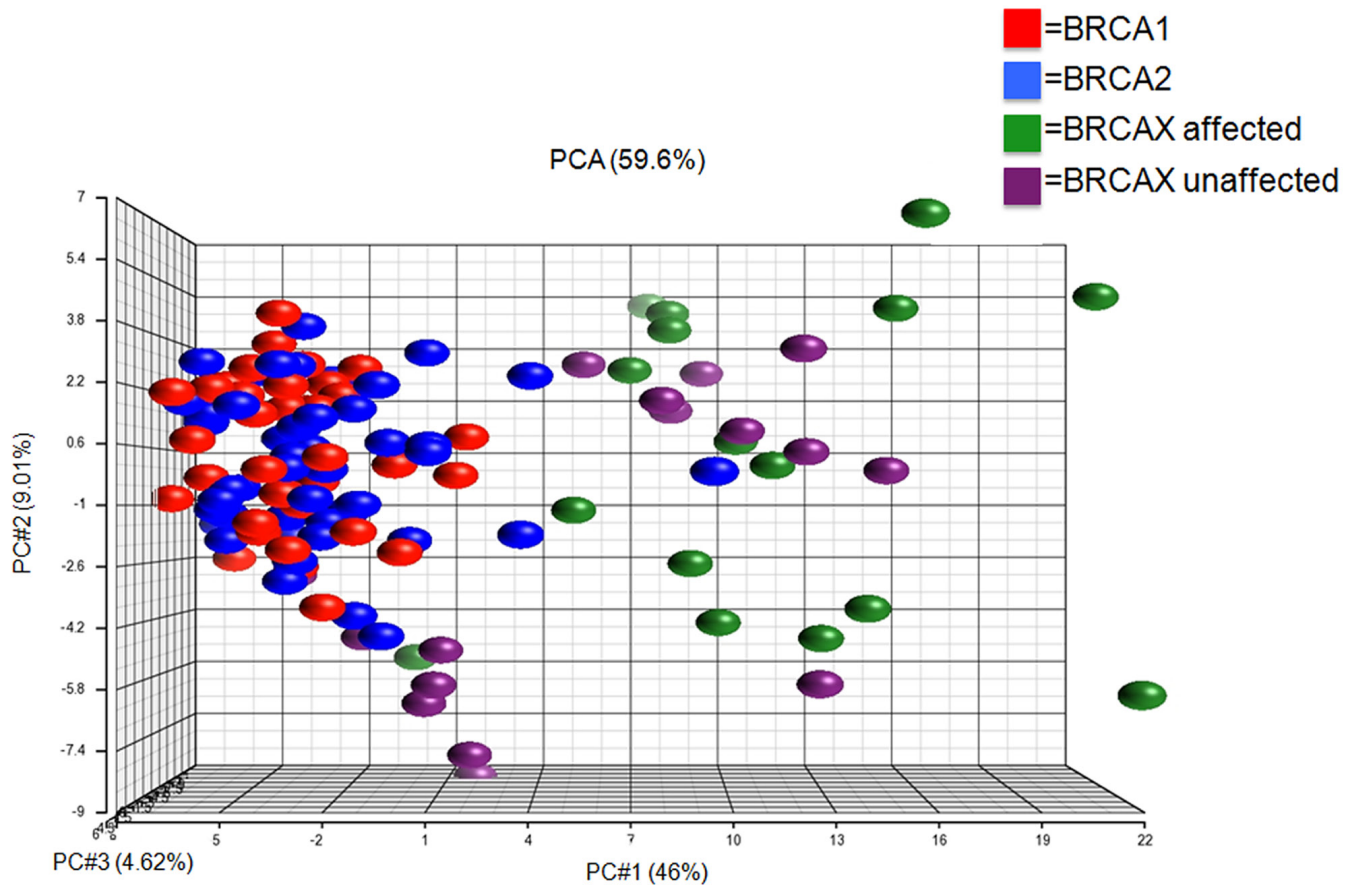
Principal component analysis (PCA) on lymphoblastoid cell lines Unsupervised classification of the groups using a combination of PC1, PC2 and PC3. Distance between dots is a dimensional measure for the similarity of the expression profiles of the samples (red: *BRCA1*, blue: *BRCA2*, green: BRCAX unaffected and purple: BRCAX affected).

Principal component analysis (PCA) of these 95 transcripts identified as differentially expressed among *BRCA1*-carriers (n=36), *BRCA2*-carriers (n=49), unaffected BRCAX (n=16) and affected BRCAX (n=16) individuals is presented in Figure 1. The first three principal components of transcriptional variation accounted for 59.6 % of the total variance. PCA on the full dataset showed that the PC1 component accounted for 46 % of the variance, which is highly informative, while PC2 was also informative compared to the variance explained in the randomized data set.

**Figure 2:**
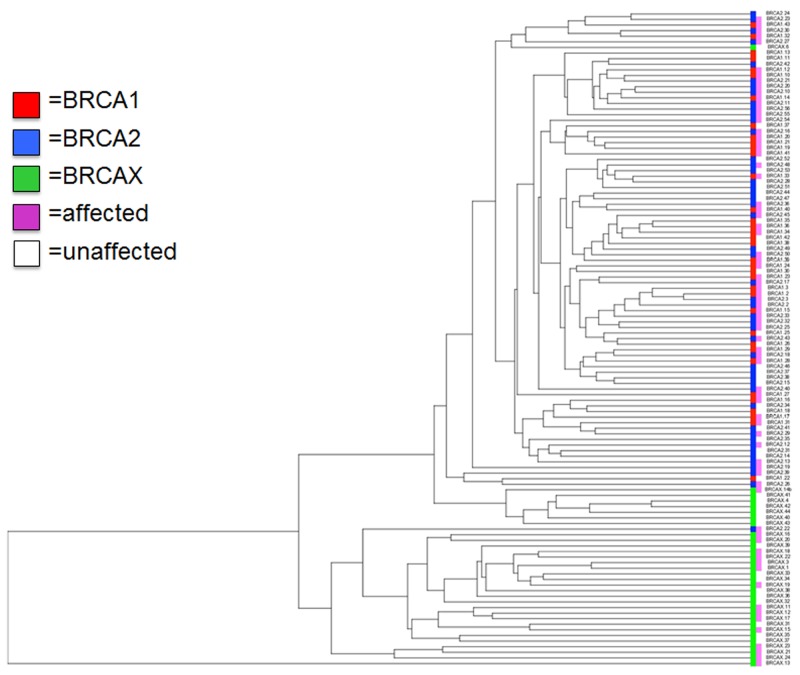
Hierarchical clustering of the 95 transcripts differentially expressed Unsupervised LCLs classification based on the significantly and differentially expressed transcripts measured by RNA-sequencing using bonferroni corrected p-value <0.01. Color bar represents each of our groups (red: *BRCA1*, blue: *BRCA2*, Green: BRCAX) and status of disease (pink: affected and white: unaffected).

Unsupervised hierarchical clustering of all *BRCA1*, *BRCA2* and BRCAX individuals was then performed using the 95 significant transcripts. As illustrated in Figure 2, gene expression levels of these significant transcripts allowed to discriminate distinctly *BRCA1/2* from BRCAX (unaffected and affected) individuals. However, it was not possible to segregate *BRCA1* from *BRCA2* individuals as well as affected from unaffected BRCAX individuals. In addition, when considering *BRCA1* and *BRCA2* individuals, no specific clustering could be observed based on gene mutation or the status of the disease. Intra-group variance analysis using gene expression data was performed by Principle component analysis (PCA) for patients with the BRCA1 R1443X mutation (22 patients) and BRCA2 8765delAG mutation (44 patients). We did not find significance of BRCA1 R1443X and BRCA2 8765delAG mutation from their respective BRCA1 and BRCA2 subgroup. Thus, grouping of different mutations in BRCA1 or BRCA2 subgroup is justified (data not shown).

The ANOVA analysis followed by conservative post hoc Scheffé test, which is appropriate for comparing groups with unequal sample sizes, allowed to potentially identify transcripts discriminating *BRCA1*, *BRCA2* and affected BRCAX individuals from unaffected BRCAX individuals, which were used as controls in this analysis. This analysis revealed 69, 71 and 28 gene isoforms differentially expressed from BRCAX unaffected for *BRCA1*, *BRCA2* and affected BRCAX individuals, respectively (See Supplementary Table 2). It should be noted that the large majority of transcripts identified in *BRCA1*-carriers were also found in *BRCA2*-carriers.

**Figure 3:**
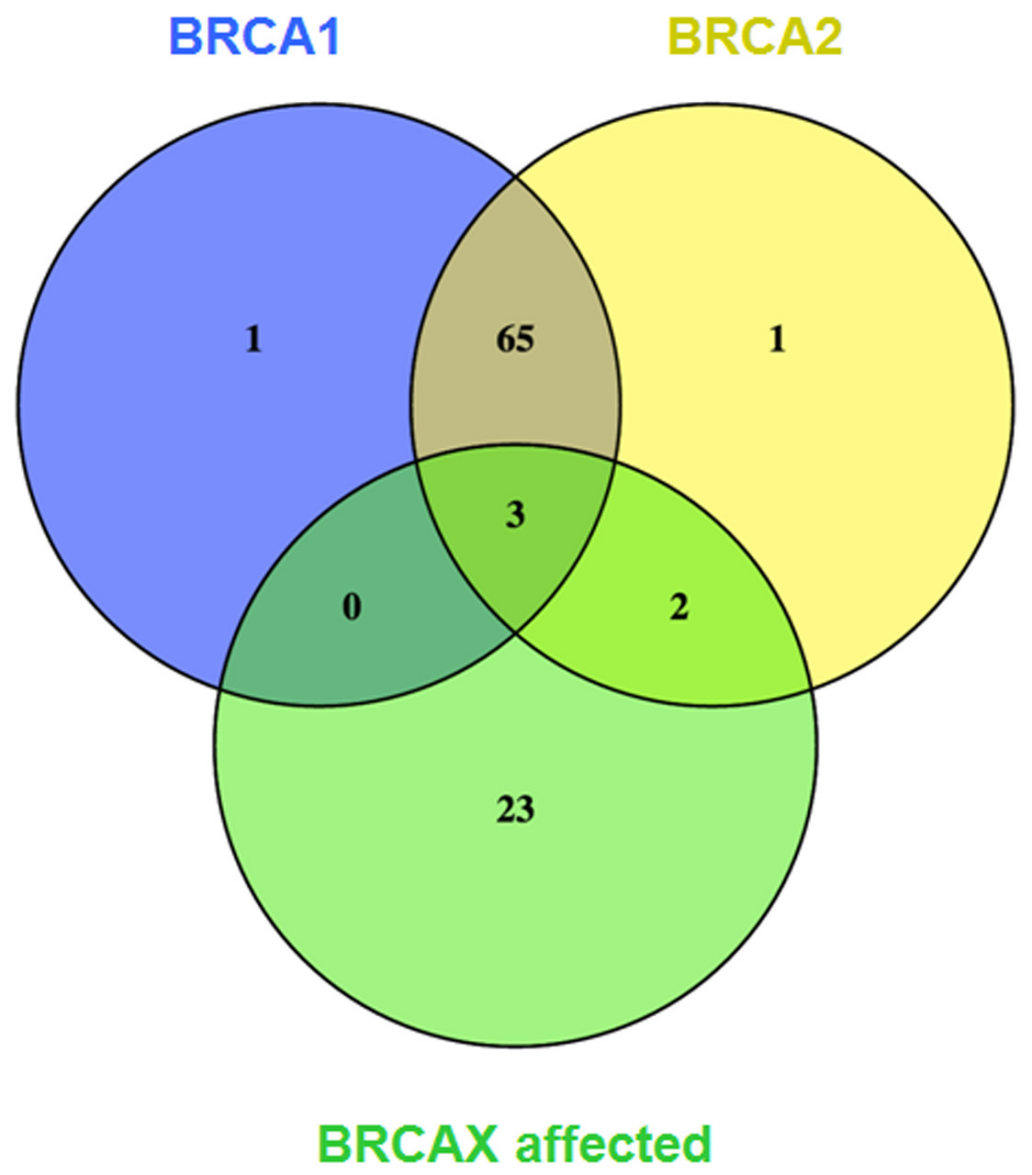
Venn diagram of significantly and differentially expressed transcripts compared with BRCAX unaffected individuals An intersectional analysis of differentially expressed transcripts compared with BRCAX unaffected was performed. The cut-off value was Bonferroni corrected p-value ≤ 0.01.

As presented in Figure 3, these transcripts were then illustrated in Venn diagrams, which showed that 3 common transcripts (3.2%) were differentially expressed in all three subgroups, when compared to unaffected BRCAX individuals. In addition, a large portion of transcripts (65: 68.4%) was commonly identified in *BRCA1* and *BRCA2* subgroups, while only 1 transcript was specifically and exclusively associated with *BRCA1*, and another one different transcript with *BRCA2*. This illustrated the similarity between *BRCA1* and *BRCA2*-carrier individuals regarding their gene expression profile. On the other hand, 23 gene isoforms were exclusively associated with affected BRCAX individuals and are not different in *BRCA1* and *BRCA2* subgroups. The name of the transcripts is presented in Supplementary Table 3.

**Figure 4:**
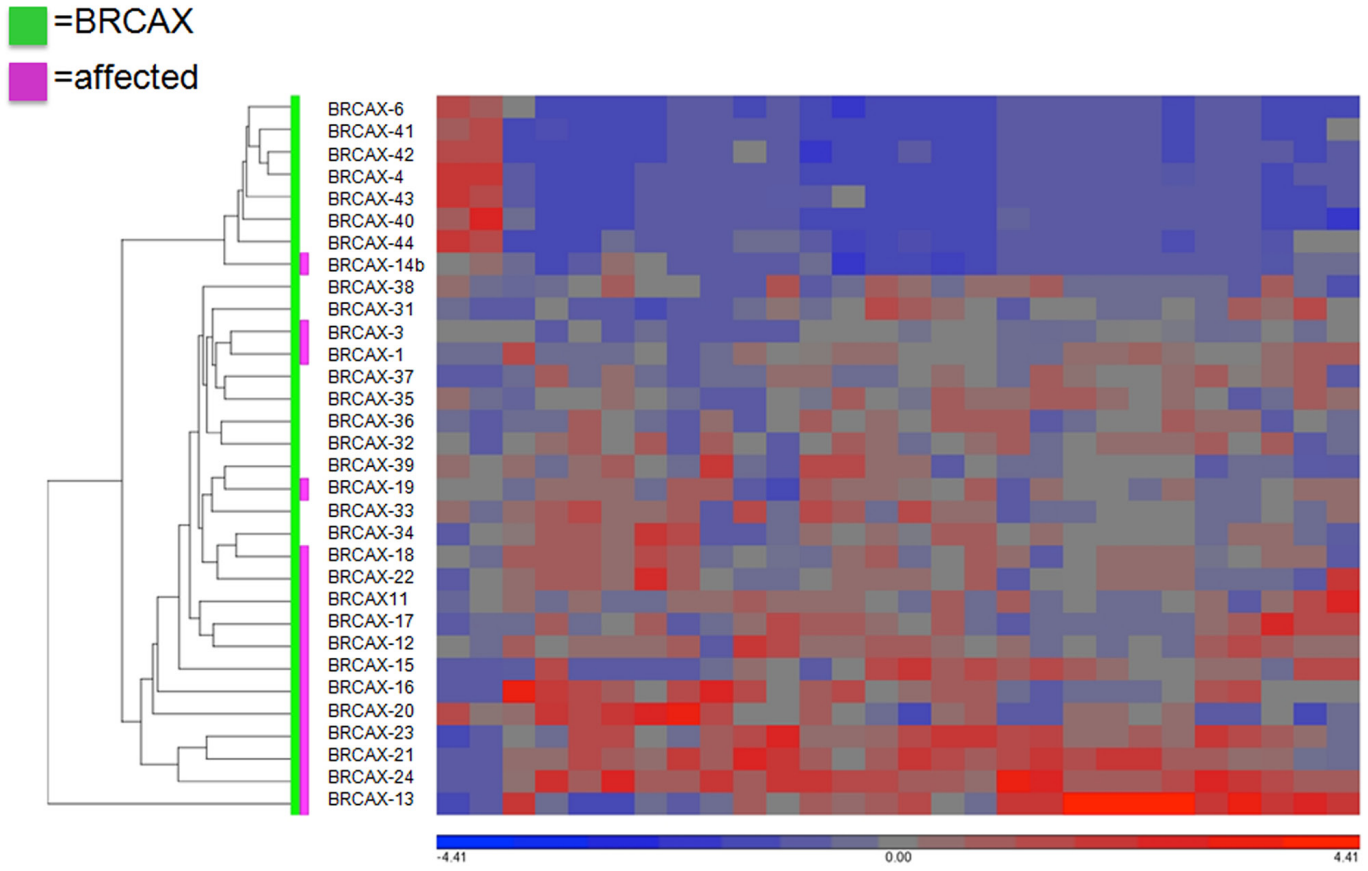
Hierarchical clustering of the 28 transcripts differentially expressed between BRCAX affected and BRCAX unaffected individuals Heat map of the TPM (Transcripts Per Million) for 32 women using Euclidean distance with average linkage.

In an attempt to further discriminate unaffected and affected BRCAX individuals, hierarchical clustering was then performed using the 28 gene isoforms discriminating both BRCAX subgroups (Figure 4). Although a much better clustering could be observed between both subgroups, these genes could not differentiate distinctly unaffected from affected BRCAX individuals, with 4 affected individuals being located among the unaffected individuals.

Further, we performed Scheffé analysis on all the 4 subgroups (*BRCA1*, *BRCA2*, unaffected and affected BRCAX) combined, this allowed us to identify specific transcripts, which are exclusively associated with each subgroup. As listed in Table 1, although no specific transcripts were specifically associated with *BRCA1* or *BRCA2* individuals, we could identify 67 transcripts specifically associated with *BRCA1/BRCA2* following combination of both subgroups and compared to BRCAX individuals. In addition, 3 and 28 transcripts showed exclusive association with unaffected and affected BRCAX subgroups, respectively.

**Table 1:** Transcripts specifically associated with each group and their corrected p-value

Transcript specifically associated with BRCA1/2 carriers when compared to BRCAX unaffected and affected individuals			
Ensembl transcript ID	HGNC symbol	Bonferroni corrected p-value	Relevant biological process
ENST00000598296	NOSIP	1.16 E-06	negative regulation of nitric-oxide synthase activity
ENST00000580799	GGA3	4.03 E-04	positive regulation of protein catabolic process
ENST00000430762	PPP3CB	1.11 E-03	positive regulation of transcription from RNA polymerase II promoter
ENST00000486593	LAMP2	1.05 E-03	regulation of protein stability
ENST00000366726	GUK1	1.18 E-03	ATP metabolic process
ENST00000438462	RTN4	1.62 E-03	Regulation of apoptotic process, cell-cell adhesion, negative regulation of cell growth
ENST00000588730	C18orf25	2.76 E-03	protein ubiquitination involved in ubiquitin-dependent protein catabolic process
ENST00000471658	PSPC1	4.22 E-03	mRNA splicing, negative regulation of transcription
ENST00000490523	EIF2AK1	3.26 E-07	negative regulation of cell proliferation, regulation of translational initiation by eIF2 alpha phosphorylation
ENST00000586868	TBCB	4.69 E-07	cell differentiation
ENST00000572932	NOMO3	1.54 E-06	carbohydrate binding
ENST00000596417	EEF2	1.09 E-06	positive regulation of translation, cell-cell adhesion, response to estradiol
ENST00000485280	RAB7A	1.99 E-06	positive regulation of protein catabolic process, regulation of autophagosome assembly
ENST00000587393	AES	3.25 E-06	negative regulation of canonical Wnt signaling pathway, negative regulation of transcription and protein binding
ENST00000593582	TRIM28	4.78 E-06	positive regulation of DNA repair, positive regulation of transcription, epithelial to mesenchymal transition, protein sumoylation and ubiquitination
ENST00000463243	HLA-DPA1	1.04 E-05	Immune process, positive regulation of interferon-gamma production
ENST00000476642	HLA-DPA1	1.04 E-05	Immune process, positive regulation of interferon-gamma production
ENST00000480481	HLA-DPA1	1.04 E-05	Immune process, positive regulation of interferon-gamma production
ENST00000483480	HLA-DPA1	1.04 E-05	Immune process, positive regulation of interferon-gamma production
ENST00000486449	HLA-DPA1	1.04 E-05	Immune process, positive regulation of interferon-gamma production
ENST00000493893	COMT	1.05 E-05	estrogen metabolic process, dopamine catabolic process
ENST00000495074	HLA-DPA1	1.04 E-05	Immune process, positive regulation of interferon-gamma production
ENST00000514979	HLA-DPA1	1.04 E-05	Immune process, positive regulation of interferon-gamma production
ENST00000524786	DEAF1	7.14 E-06	regulation of mammary gland epithelial cell proliferation, regulation of transcription
ENST00000368439	CKS1B	1.40 E-05	regulation of mitotic cell cycle
ENST00000524815	PACS1	1.83 E-05	positive regulation of protein binding, protein targeting to Golgi
ENST00000515540	BAX	3.35 E-05	DNA damage response, signal transduction by p53 class mediator resulting in cell cycle arrest, apoptotic process
ENST00000548861	RP11-603J24.9	4.07 E-05	Unknown
ENST00000529698	DGKZ	5.99 E-05	protein kinase C-activating G-protein coupled receptor signaling pathway, cell migration
ENST00000372077	VEGFA	9.13 E-05	growth factor activity, cytokine activity
ENST00000435720	PSMF1	1.51 E-04	MAPK cascade, regulation of Wnt signaling pathway and ubiquitin-protein ligase activity involved in mitotic cell cycle
ENST00000461760	STK25	1.53 E-04	positive regulation of stress-activated MAPK cascade, signal transduction by protein phosphorylation
ENST00000492277	RPL29	1.44 E-04	cell-cell adhesion, involved in nonsense-mediated decay
ENST00000236957	EEF1B2	1.68 E-04	Involved in translational elongation
ENST00000308774	TRMT112	2.13 E-04	Involved in RNA methylation and translational termination
ENST00000494862	HDLBP	2.89 E-04	cell-cell adhesion and cholesterol metabolic process
ENST00000473991	PSMD2	3.14 E-04	MAPK cascade, regulation of Wnt signaling pathway and ubiquitin-protein ligase activity involved in mitotic cell cycle, tumor necrosis factor-mediated signaling pathway
ENST00000394729	PRKCD	4.02 E-04	apoptotic process, cell cycle, negative regulation of inflammatory response
ENST00000563039	SPN	3.96 E-04	apoptotic signaling pathway, immune response, signal transduction
ENST00000406984	FTH1P15	6.76 E-04	Unknown
ENST00000585935	RAVER1	0.000677355	mRNA splicing via spliceosome
ENST00000528296	RPL8	0.000747246	cytoplasmic translation
ENST00000456311	CAD	0.000787707	cellular response to drug and epidermal growth factor stimulus
ENST00000595355	GINS2	0.000895068	double-strand break repair, mitotic DNA replication initiation
ENST00000620429	VPS11	0.000910435	positive regulation of cellular protein catabolic process, endosome to lysosome transport
ENST00000630977	VPS11	0.000910435	positive regulation of cellular protein catabolic process, endosome to lysosome transport
ENST00000352980	KAT5	0.000987787	double-strand break repair, regulation of growth and transcription
ENST00000456818	TUBA4A	0.00110052	G2/M transition of mitotic cell cycle, cytoskeleton organization
ENST00000517577	FTH1P11	0.001117274	Unknown
ENST00000591301	GNA11	0.00106912	G-protein coupled acetylcholine receptor signaling pathway, signal transduction
ENST00000523037	MRPL22	0.001163245	mitochondrial translational elongation and termination
ENST00000606722	NDUFA13	0.001321049	negative regulation of intrinsic apoptotic signaling pathway, of cell growth and transcription
ENST00000381348	LINC00634	0.001422158	Unknown
ENST00000594493	RPS11	0.001903649	Involved in nonsense-mediated decay and translation processes
ENST00000568265	TAF1C	0.002079705	positive regulation of transcription, epigenetic
ENST00000597681	MAP1S	0.002292852	apoptotic process, microtubule bundle formation
ENST00000368436	CKS1B	0.002744271	regulation of mitotic cell cycle and transcription
ENST00000537533	PTPN6	0.004221708	Regulation of apoptotic process as well as cell differentiation and proliferation
ENST00000569760	FUS	0.004169852	mRNA splicing via spliceosome and regulation of nucleic acid-templated transcription
ENST00000533397	RPL8	0.00427335	cytoplasmic translation
ENST00000443451	NCOR2	0.005527765	negative regulation of transcription
ENST00000487513	EHMT2	0.006799918	DNA methylation, regulation of transcription and DNA replication
ENST00000552600	ESPL1	0.008498519	apoptotic process, regulation of mitotic metaphase/anaphase transition and mitotic sister chromatid segregation
ENST00000543608	SPPL3	0.009823731	T cell receptor signaling pathway, positive regulation of protein dephosphorylation
ENST00000405878	XRCC6	1.10805E-07	double-strand break repair via classical nonhomologous end joining, regulation of transcription
ENST00000427834	SGSM3	1.38595E-06	Activates GTPase and binds to Rab GTPase
ENST00000537739	HDGF	4.52676E-06	Binds to the DNA and helps in cell proliferation and differentiation
**Transcript specifically associated with unaffected BRCAX when compared to BRCA1, BRCA2 and BRCAX affected individuals**			
ENST00000405878	XRCC6	1.10805E-07	double-strand break repair via classical nonhomologous end joining, regulation of transcription
ENST00000427834	SGSM3	1.38595E-06	cell cycle arrest, regulation of Rab protein signal transduction
ENST00000537739	HDGF	4.52676E-06	cell proliferation, regulation of transcription and signal transduction
**Transcript specifically associated with affected BRCAX when compared to BRCA1, BRCA2 and BRCAX unaffected individuals**			
ENST00000419477	YWHAZ	0.001164657	regulation of apoptotic process, cell-cell adhesion and establishment of Golgi localization
ENST00000539269	CARS2	0.002107185	cysteinyl-tRNA aminoacylation
ENST00000436614	ZNF687	5.67433E-07	regulation of transcription
ENST00000237837	FGF23	6.98876E-06	MAPK cascade, fibroblast growth factor receptor signaling pathway, regulation of transcription
ENST00000452722	CADM1	6.01183E-05	apoptotic process, regulation of cytokine secretion
ENST00000459748	RP11-466H18.1	0.000157558	Unknown
ENST00000460469	NMD3	0.000153464	protein transport
ENST00000562465	CDAN1	0.000146598	chromatin assembly, negative regulation of DNA replication
ENST00000495645	CHPF2	0.000410748	chondroitin sulfate biosynthetic process
ENST00000377861	PCDH9	0.000632184	homophilic cell adhesion via plasma membrane adhesion molecules
ENST00000415265	WDR6	0.000658126	cell cycle arrest, negative regulation of cell proliferation
ENST00000552588	RPL18	0.00085376	Involved in nonsense-mediated decay and translational initiation
ENST00000374752	ACAD8	0.001441532	lipid metabolic process, regulation of transcription
ENST00000449683	ATP5J2	0.001764478	ATP biosynthetic process
ENST00000513391	OCIAD1	0.001957078	protein binding
ENST00000547276	HNRNPA1	0.002375206	fibroblast growth factor receptor signaling pathway, gene expression, mRNA splicing
ENST00000525085	NDUFC2	0.003100189	mitochondrial electron transport, NADH to ubiquinone
ENST00000500813	DCTD	0.003245736	nucleotide biosynthetic process
ENST00000612832	ARHGAP21	0.004173962	organelle transport along microtubule, Golgi organization
ENST00000535413	MLEC	0.004822683	carbohydrate metabolic process, protein folding
ENST00000498022	NAGK	0.004945162	UDP-N-acetylglucosamine biosynthetic process
ENST00000444034	MED12	0.005486952	canonical Wnt signaling pathway, intracellular steroid hormone receptor signaling pathway, regulation of transcription
ENST00000522754	NCALD	0.006089983	calcium-mediated signaling
ENST00000552819	PCBP2	0.002187131	mRNA metabolic process, mRNA splicing, defense response to virus
ENST00000528413	IRF7	0.002697978	cellular response to DNA damage stimulus, interferon-gamma-mediated signaling pathway, regulation of transcription and immune response
ENST00000405878	XRCC6	1.10805E-07	double-strand break repair via classical nonhomologous end joining, regulation of transcription
ENST00000427834	SGSM3	1.38595E-06	cell cycle arrest, regulation of Rab protein signal transduction
ENST00000537739	HDGF	4.52676E-06	IRE1-mediated unfolded protein response, cell proliferation, regulation of transcription and signal transduction

Of interest, out of 67 transcripts specifically associated with *BRCA1/BRCA2* subgroups combined, 19 transcripts (28%) were involved in DNA repair, cell proliferation, apoptosis and cell cycle, 32 (48%) different transcripts exert an action in transcription, translation as well as mRNA and protein metabolic processes, while 10 transcripts (15%) were involved in immune processes. Regarding the 28 transcripts exclusively associated with BRCAX affected individuals, more than 28% were involved in DNA repair/proliferation/apoptosis/cell cycle mechanisms, and approximately 43% (12 transcripts) were implicated in transcription and translation-related processes. It should be noted that *XRCC6*, *SGSM3* and *HDGF* transcripts were associated with *BRCA1/BRCA2*, BRCAX unaffected and BRCAX affected individuals given that their expression was differentially expressed between all three subgroups. The expression of *SGSM3* and *HDGF* was validated by qPCR in BRCAX subgroup to differentiate affected from non-affected patients (Supplementary Figure 1). The expression level of these genes was also checked in different mammalian breast cancer cell lines. Further, ANOVA analysis highlighted that *H3F3B* was differentially expressed in *BRCA1* and *BRCA2* subgroups, which was also evaluated by qPCR (Supplementary Figure 1).

The 85 genes associated with the 95 significant transcripts identified as differentially expressed between *BRCA1*, *BRCA2* and BRCAX (unaffected or affected) individuals were then submitted for pathway and molecular function analyses. Using Ingenuity Pathway analysis, enrichment of several canonical pathways and functions were identified. It should be noted that mapped genes can be classified in more than one biological process or metabolic process.

Moreover, as described in Table 2, cell death and survival (top p-value: 2.46 × 10^−5^ with 34 molecules), cellular function and maintenance (top p-value: 6.77 × 10^−5^ with 25 molecules), cell cycle (top p-value: 7.41 × 10^−5^ with 15 molecules), post-translational modification (top p-value: 2.03 × 10^−4^ with 11 molecules) as well as cell morphology (top p-value: 2.07 × 10^−4^ with 17 molecules) represent the top overrepresented functions associated with these 85 genes. In addition, organismal injury, cell signaling, cell cycle and cell death represent the top networks associated with the whole gene set. Of great interest, these genes were also associated with Cancer and Organismal Injury and Abnormalities (Table 2).

**Table 2:** Overrepresented functions, network and diseases for significantly and differentially expressed transcripts

Molecular and cellular functions	Top p-value*
Cell Death and Suvival [34]	2.46E-05
Cellular Function and Maintenance [25]	6.77E-05
Cell Cycle [15]	7.41E-05
Post-Translational Modification [11]	2.03E-04
Cell Morphology [17]	2.07E-04

Top Networks	Score
Organismal Injury and Abnormalities, Renal and Urological Disease, Embryonic Development	61
Psychological Disorders, Cancer, Cell Cycle	29
Cell Signaling, Cell-To-Cell Signaling and Interaction, Cell Death and Survival	25
Cell Cycle, Cancer, Organismal Injury and Abnormalities	22
Cell Death and Survival, Cancer, Gastrointestinal Disease	15

Diseases and disorders	Top p-value*
Cancer [71]	1.45E-05
Organismal Injury and Abnormalities [73]	1.45E-05
Renal and Urological Disease [27]	1.45E-05
Neurological Disease [27]	4.17E-05
Hematological Disease [34]	1.86E-04

*Top P-value of the p-value range.

The IPA analysis was also performed using the 28 genes discriminating unaffected from affected BRCAX individuals. As presented in Table 3, BRCAX-related genes were particularly associated with Telomere Extension by Telomerase, DNA Double-Strand Break Repair by Non-Homologous End Joining as well as specific molecule degradation and biosynthesis (p-value ranging from 1.66 × 10^−4^ to 0.05). The top system development and functions represented by these genes were tissue development (top p-value: 1.28 × 10^−3^ with 5 molecules), embryonic development (top p-value: 1.28 × 10^−3^) and Immune Cell Trafficking (top p-value: 1.28 × 10^−3^) (data not shown).

**Table 3:** The most significant canonical pathways enriched for significantly and differentially expressed transcripts between BRCAX affected and BRCAX unaffected

IPA canonical enriched pathways	Number of gene in pathways	p-value
Telomere Extension by Telomerase	2	1.66E-04
N-acetylglucosamine Degradation II	1	5.13E-03
CMP-N-acetylneuraminate Biosynthesis I (Eukaryotes)	1	6.46E-03
Chondroitin and Dermatan Biosynthesis	1	7.76E-03
DNA Double-Strand Break Repair by Non-Homologous End Joining	1	1.78E-02
Isoleucine Degradation I	1	1.78E-02
Valine Degradation I	1	2.29E-02
tRNA Charging	1	4.90E-02

Analyses were performed using genes associated with significantly and differentially expressed transcripts between BRCAX affected and BRCAX unaffected using bonferroni correct p-value <0.01.

In addition, cell-to-cell signaling and interaction, cellular development, cellular growth and proliferation, cellular movement and lipid metabolism (top p-value at 1.28 × 10^−3^) represented the enriched functions (Table 4). As also described in this table, IPA analysis revealed that these genes were involved in networks such as “Cell death and survival” as well as “Connective tissue disorders and metabolic diseases” with 38 and 26 molecules, respectively.

**Table 4:** Overrepresented functions, network and diseases for significantly and differentially expressed transcripts between BRCAX affected and BRCAX unaffected

Molecular and cellular functions	Top p-value*
Cell-To-Cell Signaling and Interaction [4]	1.28E-03
Cellular Development [7]	1.28E-03
Cellular Growth and Proliferation [4]	1.28E-03
Cellular Movement [2]	1.28E-03
Lipid Metabolism [1]	1.28E-03

Top networks	Score
Cell Death and Survival, Cell Morphology, Hair and Skin Development and Function	38
Connective Tissue Disorders, Metabolic Disease, Nutritional Disease	26
Cellular Assembly and Organization, DNA Replication, Recombination, and Repair, Nucleic Acid Metabolism	2

Diseases and disorders	Top p-value*
Connective Tissue Disorders [3]	2.37E-05
Metabolic Disease [3]	2.37E-05
Nutritional Disease [2]	2.37E-05
Skeletal and Muscular Disorders [4]	2.37E-05
Cancer [26]	5.46E-04

* Top P-value of the p-value range.

The number of genes involved in the process is given in parentheses.

Analyses were performed using genes associated with significantly and differentially expressed transcripts between BRCAX affected and BRCAX unaffected using bonferroni correct p-value <0.01.

Moreover, “cancer” (top p-value: 5.46 × 10^−4^ with 26 molecules) represented the disease associated with the highest number of molecules. Altogether, these analyses revealed enrichment of several pathways and functions involved in key mechanisms required for carcinogenesis development.

## DISCUSSION

In this study, we analyzed the genome-wide transcription profile observed in LCLs immortalized from high-risk breast cancer families. To our knowledge, this is the first study describing clustering of *BRCA1*, *BRCA2* and unaffected/affected BRCAX individuals based on their whole gene expression profile observed in corresponding LCLs.

The reliability of using LCLs from affected individuals for a given disease with regard to expression studies or splicing signatures has already been established [18-28]. A particular study conducted by Hussain and colleague concluded that LCLs were a good reflection of isolated lymphocytes given their close resemblance at the genetic and phenotypic levels to parent lymphocytes and were a valuable resource for studies regarding genotype-phenotype interactions [39] and inter-individual variations associated with various diseases and disorders such as cancer or infectious disease [40-42]. In addition, peripheral blood mononuclear cells (PBMCs) have also been used to investigate the links between DNA damage response, immunity and cancer [43] and to study the early stages of breast cancer development on gene expression patterns [44].

As described in other studies [45-47], we used ANOVA analysis of variance with the Scheffé multiple post-hoc test to identify transcripts specifically regulated in *BRCA1*, *BRCA2* as well as in unaffected and affected BRCAX individuals. Using gene expression data of the 95 significant gene isoforms, our clustering results allowed discrimination of *BRCA* subgroups, particularly *BRCA1/2* from BRCAX individuals.

This is in agreement with other studies in which gene expression in LCLs was successfully used for clustering analysis in several diseases including autism spectrum disorders and spinocerebellar ataxia (*SCA28*) [48, 49]. Moreover, LCLs served as a model system to assess genotype–phenotype relationships in human cells, including studies for quantitative trait loci influencing levels of individual mRNAs and responses to drugs and radiation [50-53], as well as regarding the haploinsufficiency effects of various *BRCA1* mutation [54].

Over the last decade, several investigations clustered breast tumors based on single gene expression levels as well as their gene/splicing expression profile or molecular and clinical characteristics. The first correlation between the tumor phenotypic diversity (histopathological and clinical characteristics) and gene expression patterns was demonstrated in 2000 by Perou and colleague [55-58]. Van’t Veer et al. conducted clustering analyses of breast tumors based on their gene expression profile and determined a predictive signature of metastases development (poor prognosis) in patients without tumoral cells in local lymph node at diagnosis and established a specific signature of *BRCA1* tumors [56]. Clustering analyses of breast tumors based on whole gene expression revealed familial aggregation of BRCA-related tumors and of specific molecular subtypes including Basal, *HER2*-enriched, Luminal A and B as well as Normal-like and sporadic tumors [38, 55-57, 59-70]. In addition, alternative splicing expression profile was also successfully used in several studies aiming to discriminate subtypes of breast tumors [71-73], and specific gene expression profiles have also been identified for *BRCA1*, *BRCA2* and *CHEK2*-associated breast tumors [62, 74].

To our knowledge, the only clustering analysis involving LCLs in breast cancer classification distinguished BRCA1 carrier from non-carrier individuals [75], in which 133 genes were found to be differentially expressed between *BRCA1*-mutated and non-carriers. However, hierarchical clustering of these genes did not result in an accurate discrimination between both subgroups. Of these 133 genes identified by Vuillaume *et al.* [75], the *RPL29* and *PSMF1* genes have also been identified in our comparison between *BRCA1/2* and BRCAX individuals. *RPL29* is a ribosomal protein, involved in RNA interaction and protein synthesis, while *PSMF1* gene encodes a proteasome inhibitor protein involved in protein folding and degradation [76, 77].

We then compared our results with GTEx Portal database, which contains normalized expression data from RNA sequencing for each gene and transcripts for different types of tissues. The normalization of expression was done by similar method for all databases. The expression values for genes from EBV transformed lymphocytes are available, and the normalized expression values in this database are similar with the ones we obtained [78]. In addition, we performed correlation between TPM value and FPKM value by doing a regression analysis using the values for each sample in the analysis and we found significant correlation between both of them.

We also compared our gene lists with the lists for up and down regulated genes associated with breast cancer using BioXpress, a curated gene expression and disease association database using RNA sequencing from TCGA database [79]. A certain number of our significant genes were common. Indeed, there were 5 genes that were present in our list and in the up regulated list for breast cancer (*RAP2C*, *SPN*, *GINS2*, *ESPL1* and *IRF7*). Regarding the list for down regulated genes associated with breast cancer, 3 genes were also present in our list (*HLA-DPA1*, *PCDH9* and *NCALD*).

Among the highest significant genes associated with *BRCA1/2* subgroups, some of them (p-values ranging from 1.1 × 10^−7^ to 4.5 × 10^−6^) namely *NOSIP*, *EIF2AK1*, *TBCB*, *XRCC6*, *SGSM3* and *HDGF* are involved in key mechanisms implicated in carcinogenesis susceptibility.

The expression of the *NOSIP* gene was upregulated and *EIF2AK1* and *TBCB* were downregulated in *BRCA1/2* individuals when compared to BRCAX subgroups. The eNOS Interacting Protein *NOSIP* was identified as an interacting protein of the endothelial isoform of nitric oxide synthase (eNOS) to enhance its translocation to intracellular membrane [80].

The *EIF2AK1* gene is involved in the modulation of the basal hepatic endoplasmic reticulum stress tone [81]. Although no information links this gene to breast cancer, it has been demonstrated in a mouse xenograft model of human breast cancer that an activator of *EIF2AK1* protein was associated with tumor growth inhibition compared with vehicle [82]. Thus, its downregulation in *BRCA1/2* individuals could promote tumor development.

Regarding *TBCB*, this protein is involved in regulation of axonal growth and microtubule functional diversity and dynamics [83]. This protein was shown to be overexpressed and phosphorylated in breast tumors [84]. Therefore the effect of its decreased expression in *BRCA1/2* individuals remains to be investigated.

Of great interest, specific expression levels of *XRCC6*, *SGSM3* and *HDGF* genes were also associated with BRCAX individuals (unaffected and affected). These genes are involved in DNA repair as well as in the regulation of cell cycle, cell proliferation and transcription, and were differentially expressed between *BRCA1/2*, unaffected BRCAX and affected BRCAX individual. These genes showed the highest expression in affected BRCAX and the lowest expression in *BRCA1* and *BRCA2* subgroups. Indeed, a similar and progressive pattern of expression values for all three genes was observed between subgroups (BRCAX affected > BRCAX unaffected > *BRCA1/2* individuals) as presented in Supplementary Table 2. Futhermore, the study highlighted few genes which could discriminate *BRCA1* from *BRCA2* subgroup, amongst them the highest difference was depicted by *H3F3B.*

*XRCC6* gene encodes the Ku70 protein, which is a component of the non-homologous end joining (NHEJ) DNA repair pathway. This pathway is an alternative mechanism to homologous recombination (HR) repair pathway involved in double-strand break (DSB) repair in mammalian cells [85]. Defect or variation of expression of *NHEJ* genes such as *XRCC6*, might escape cell cycle checkpoint surveillance and could lead to suboptimal DNA repair and subsequently to accumulation of DNA damage and carcinogenesis initiation [86-88]. Given the key roles of BRCA1/2 in HR repair pathway [89], defective activity of BRCA1/2 proteins found in some individuals combined with low expression of *NHEJ*-associated genes could likely increase the accumulation of DNA damage in BRCA1/2 individuals. Indeed, this low expression of Ku70 was previously observed in BRCA1-deficient cell lines [90]. On the other hand, the high expression of *XRCC6* in BRCAX individuals affected with breast cancer remains to be elucidated. Moreover, polymorphisms in *XRCC6* gene have been shown to increase breast cancer susceptibility as well as other types of cancer [91-96].

SGSM3 is a member of the small G protein signaling modulators, which is associated with small G protein coupled receptor signal transduction pathway [97]. The human SGSM3 proteins were demonstrated to coprecipitate with RAP and RAB subfamily members of the small G protein superfamily. Therefore it has been suggested that the SGSM family members exert a role as modulators of the small G protein RAP and RAB-mediated neuronal signal transduction and vesicular transportation pathways [97]. The only information in the literature associating this protein with breast cancer described a decrease of *SGSM3* mRNA in breast cancer tissue compared to normal tissue [98], which is in contrast with the significant increase of expression in LCLs of BRCAX affected individuals observed in our study. However, in the study performed by Nourashrafeddin *et al.* using basic RT-PCR method and visualization on agarose gel [98], *SGSM3* was not detected in normal and cancerous tissues, illustrating the very low expression of this gene in breast tissues. It should be noted that a polymorphism (rs17001868) found in the *SGSM3* gene has been associated with mammographic dense areas of the breast [99], which represents a factor of breast cancer risk [100-102]. *SGSM3* has also been associated with hepatocellular carcinoma [103].

The Hepatoma-derived growth factor (*HDGF*) is now recognized as a breast cancer-associated gene and promotes the epithelial-mesenchymal transition (EMT) [104]. EMT is a hallmark of many cancers characterized by an increased cell invasion, which enhances the initial phase of metastatic progression [105, 106]. HDGF is overexpressed in several types of cancers including breast cancer cell lines and tissues and correlates with poor prognosis [104, 107-112]. Blockade of HDGF using a specific antibody results in the inhibition of malignant features and EMT of breast cancer cells [104]. Thus, its overexpression in breast cancer tissues is in concordance with our results demonstrating the overexpression of *HDGF* in LCLs of BRCAX individuals affected with breast cancer. Hence, this protein could be considered as a prognostic factor for tumor metastasis and recurrence.

H3 Histone Family Member 3B (*H3F3B*) is part of core histone molecule and has a role in gene regulation, DNA repair, DNA replication and chromosomal stability. Mutations in *H3F3B* gene have been associated with several cancers including brain cancer, giant cell tumor of bone and colorectal cancer [113-115]. Overexpression of this gene is also associated with colorectal cancer. In breast cancer, the copy number of the chromosome carrying this gene is significantly high [116], which is further confirmed by the data from the human protein atlas data.

In addition to *XRCC6*, *SGSM3* and *HDGF* genes as described above, *ZNF687* and *FGF23* genes were also associated with and upregulated in BRCAX affected individuals when compared to unaffected individuals.

*ZNF687* encodes a zinc finger protein and represents an important regulator of skeletal development and maintenance [117]. Overexpression of *ZNF687* has been observed in tumor tissue of individual giant cell tumor of bone associated with Paget disease of bone, and this high expression is also observed in the peripheral blood of patients affected with Paget disease [118]. The role of *ZNF687* in breast cancer is unknown.

As to fibroblast growth factor 23 (FGF23), it is a binding partner of Klotho proteins for endocrine signaling through the action of FGFRs. These FGFR receptors are involved in several mechanisms such as regulation of cell survival, proliferation, differentiation and motility during embryogenesis as well as tissue homeostasis and carcinogenesis [119-122]. Indeed, FGF23 signaling promotes proliferation in myeloma cells [123], while increase of FGF23 levels in serum were observed in cancer patients, and were also elevated in patients with non-cancerous diseases, such as hypophosphatemic rickets and chronic kidney diseases [124].

Considering all significant genes identified following ANOVA analysis of gene expression data observed in four *BRCA* subgroups, several interesting pathways seem to be affected by the regulation of specific genes. Among these pathways, *EIF2* signaling, *14-3-3*-mediated pathway and *mTOR* signaling are particularly significant and relevant to breast cancer. Moreover, both the *EIF2* (through the activation of the *PI3K* pathway) and 14-3-3-mediated signaling cascades regulate the *mTOR* pathway [125, 126], which is involved in the response to hormones and growth factor stimulation and is well known to exert a significant role in tumor cell growth and proliferation as well as in breast cancer development [127] and references therein.

On the other hand, cell death and survival, cellular function and maintenance as well as cell cycle represent the highest enriched functions, while cancer remains among the diseases/disorders having the highest p-value, which is associated with a high number of genes involved in cancer-related pathways.

Taken together, in the present study, we compared gene expression profiles in lymphoblastoid cell lines in *BRCA1*- and *BRCA2*- carriers as well as BRCAX affected and unaffected individuals from high-risk breast cancer families in order to determine specific markers which could be of great relevance for further studies. Indeed, several transcripts have been identified as potential valuable markers of interest for breast cancer, and deserve further analysis.

## MATERIALS AND METHODS

### Ascertainment of high-risk families

Recruitment of high-risk French Canadian breast and ovarian cancer families started in 1996 as a large interdisciplinary research program designated INHERIT BRCAs [12]. The High risk group is defined as families with a history of breast cancer with at least 3 cases in 1^st^ degree relative or 4 cases in 2^nd^ degree relative, the full selection criteria have been published previously [12]. Patients were screened for deleterious mutations in *BRCA1* and *BRCA2* genes. The *BRCA* testing was done by complete sequencing of the *BRCA1/2* gene by using primers in both directions (forward and reverse). Confirmation was done by Myriad Laboratories. A subset of 96 high-risk families with no deleterious mutation in *BRCA1* or *BRCA2* were recruited (BRCAX families) as described elsewhere [128, 129]. For the purpose of this study, carriers of a *BRCA1* or *BRCA2* mutation were selected. As for the BRCAX families, the youngest available breast cancer case in the family was selected, along with the oldest non-affected sister. All unaffected women were post-menopausal. All individuals provided their written informed consent in order for their genetic material to be part of a biobank (Dr J. Simard, director). The age range of affected *BRCA1, BRCA2* and BRCAX were 23-65, 29-72 and 35-70 respectively. For unaffected *BRCA1, BRCA2* and BRCAX the age range was 35-66, 37-77 and 41-86 respectively.

#### Cell line immortalization and RNA extraction

Lymphocytes (LCLs) were isolated and immortalized from 7 to 9 mL of blood samples from breast cancer individuals using Epstein-Barr virus in 15% RPMI medium as previously described [128, 130, 131]. Total RNA was extracted from LCLs using TRI Reagent (Molecular Reasearch Center Inc, Cincinnati, OH, USA) according to the manufacturer’s instructions as described previously [128]. The viral strain, number of passage and conditions for cell lines were kept identical to avoid bias in gene expression [133].

#### RNA-seq experiments

The quality of RNA samples was evaluated with an Agilent Bioanalyzer 2100 to determine the RIN (RNA Integrity) score using the Agilent RNA 6000 Nano chip and reagents. Samples with a RIN score >7 were retained and converted to cDNA with the Illumina RNA seq kit for sequence library preparation based on the Illumina TruSeq RNA Sample Preparation protocol. The final libraries were pooled in triplicate and then sequenced on an Illumina HiSeq 2000 at the McGill University and Génome Québec Innovation Centre.

Raw reads were trimmed for length (n>=32), quality (phred33 score >= 30) and adaptor sequence using fastxv0.0.13.1. Trimmed paired-end reads (read length: 100 bp) were aligned to the hg19 human reference genome using Tophat version v1.4.0 [132]. The resulting alignment file was indexed using samtools v0.1.18. Raw, trimmed and aligned read numbers were retrieved after alignment to determine the quality of the sequence data. GATK (v1.0.5777) [134] was then used to compute the coding sequence coverage for each sample. Raw read counts and normalized read counts (in transcript per million, TPM) were obtained using the Kallisto v0.43.0 quant command [135] with the default parameter on the GRCh38.rel79 version of the human transcriptome, while Pearson correlation values were obtained pairwise for each sample using R v2.12.0. Differential gene expression was determined using edgeRv2.2.6 [136] on R v2.12.0 and DESeqv1.6.1 [137] on R v2.14.0. Transcript differential expression was performed using cuffdiff v1.3.0. A gene ontology analysis was then launched on gene and transcript differential expression results using goseqv1.2.1 [138] on R v2.12.0. Finally, UCSC compatible wiggle tracks were generated using FindPeaks v4.0.16.

#### Statistical analysis

Statistical analyses were carried out using the R Package v3.3. In regard to mRNA levels, One-factor analysis of variance (ANOVA) was performed to compare the breast cancer subgroups. First a model was fitted using the lm function from the stats package then the ANOVA analysis was performed with the Anova command from the car package [139-140]. Bonferroni correction was performed with the p.adjust function from the stats package using “BH”, “BY” and “bonferroni” methods and statistically significant differences were considered at p < 0.01 for the “bonferroni” method [140]. The Scheffé test was carried out with the scheffé test function from the agricolae R package for post-hoc analysis for comparisons between two of the multiple groups [141]. We performed intra-group variance analysis using gene expression data of patients with the *BRCA1* R1443X mutation and *BRCA2* 8765delAG mutation by Principle component analysis (PCA).

#### Pathways, network and clustering analyses

Partek Genomics Suites® software package (copyright © 2009 Partek Incorporated. St. Louis, MO) was used for hierarchical clustering using the default setting (Euclidean dissimilarity and average linkage method) as well as for Principal component analysis (PCA).

Identification of overrepresented pathways, functions and gene-associated diseases were performed using QIAGEN’s Ingenuity® Pathway Analysis (IPA®, QIAGEN Redwood City, www.qiagen.com/ingenuity) software. Default settings in IPA for expression dataset analyses were used for gene list functional analysis. Gene lists were uploaded using NCBI Entrez gene IDs or gene symbols and submitted for IPA Core Analysis. IPA calculates *p*-values that reflect the statistical significance of association between the genes and the networks by the Fisher’s exact test. *P*-value ≤ 0.05 were considered significant.

## SUPPLEMENTARY MATERIALS FIGURES AND TABLES








