# Supplementary material for: Transcriptional signature of lymphoblastoid cell lines of BRCA1, BRCA2 and non-BRCA1/2 high risk breast cancer families
Source: Oncotarget. 2017 Aug 12;8(45):78691–712. doi: 10.18632/oncotarget.20219 (PMC5667991; doi:10.18632/oncotarget.20219)
Supplement: Supplementary file 1 [file oncotarget-08-78691-s001.pdf]

Transcriptional signature of lymphoblastoid cell lines of *BRCA1*, *BRCA2* and non-*BRCA1/2* high risk breast cancer families

SUPPLEMENTARY MATERIALS

Supplementary Table 1: Characteristics of BRCA- individuals

See Supplementary File 1

Supplementary Table 2: Annova test results

See Supplementary File 2

Supplementary Table 3: Transcripts associated with each subgroup

See Supplementary File 3

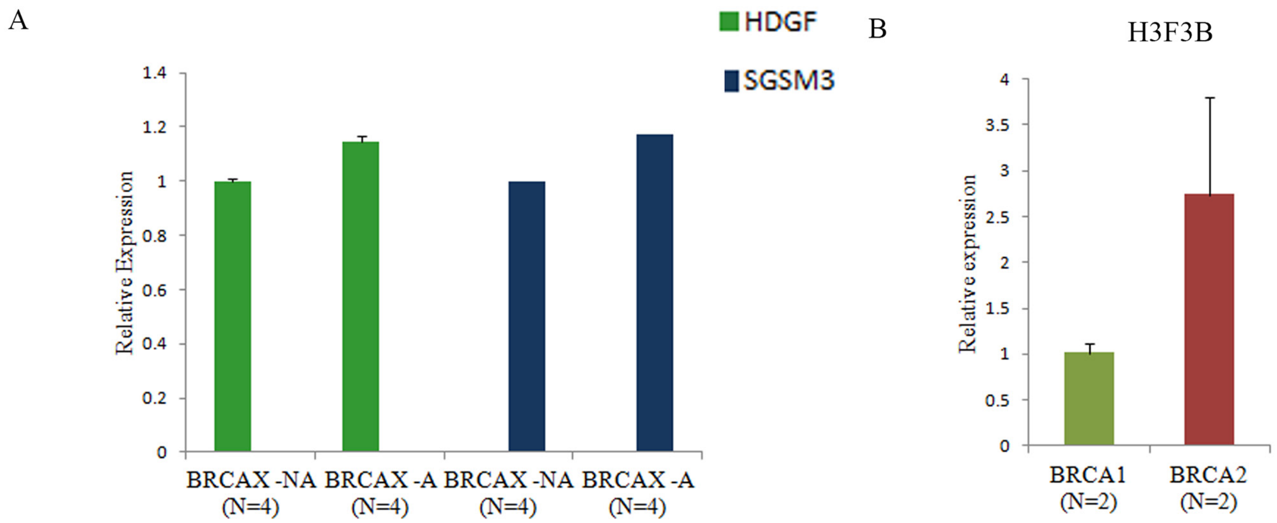

Supplementary Figure 1: Validation of differentially expressed genes in different subgroups by qPCR. Expression level of *HDGF* and *SGSM3* in BRCAX-Unaffected (BRCAX-NA) and BRCAX –Affected subgroup **(A)** and *H3F3B* in BRCA1 and BRCA2 subgroups. The graph is the representation of 3 independent experiments. The low level of difference in the expression level in BRCAX subgroup is reproducible in all the experiments.
