# Supplementary material for: Transcriptional signature of lymphoblastoid cell lines of BRCA1, BRCA2 and non-BRCA1/2 high risk breast cancer families
Source: Oncotarget. 2017 Aug 12;8(45):78691–712. doi: 10.18632/oncotarget.20219 (PMC5667991; doi:10.18632/oncotarget.20219)
Supplement: Supplementary file 2 [file oncotarget-08-78691-s002.docx]

| **Supplementary Table 1: Characteristics of BRCA- individuals** | | | |
| --- | --- | --- | --- |
| **Individuals ID** | **Cancer (age at diagnosis)** | **Deleterious mutation in *BRCA1* and *BRCA2*** | **Relationship** |
| BRCA1-10 | Breast cancer at 30 and 43 year | B1-R1443X | With BRCA1-12 |
| BRCA1-11 | Unaffected | B1-R1443X | - * |
| BRCA1-12 | Breast cancer at 39 and 49 year | B1-R1443X | With BRCA1-10 |
| BRCA1-13 | Unaffected | B1-R1443X |  |
| BRCA1-14 | Ovarian cancer at 59 and breast cancer at 61 year | B1-R1443X | With BRCA1-15 |
| BRCA1-15 | Breast cancer at 38 and 60 year | B1-R1443X | With BRCA1-14 |
| BRCA1-16 | Breast cancer at 57 year | B1-3705insA | With BRCA1-17 |
| BRCA1-17 | Breast cancer at 54 year | B1-3705insA | With BRCA1-16 |
| BRCA1-18 | Unaffected | B1-R1443X |  |
| BRCA1-19 | Breast cancer at 49 and 52 year | B1-R1443X | With BRCA1-20 |
| BRCA1-20 | Breast cancer at 49 year | B1-R1443X | With BRCA1-19 |
| BRCA1-21 | Breast cancer at 44 and 48 year | B1-2953del3+C |  |
| BRCA1-22 | Unaffected | B1-R1443X |  |
| BRCA1-23 | Breast cancer at 51 and 54 year | B1-R1443X |  |
| BRCA1-24 | Breast cancer at 43 and 49 year | B1-2244insA |  |
| BRCA1-25 | Unaffected | B1-2244insA | With BRCA1-26 and BRCA1-27 |
| BRCA1-26 | Unaffected | B1-2244insA | With BRCA1-25 and BRCA1-27 |
| BRCA1-27 | Breast cancer at 39 year | B1-2244insA | With BRCA1-25 and BRCA1-26 |
| BRCA1-28 | Breast cancer at 49 year | B1-R1443X |  |
| BRCA1-29 | Breast cancer at 42 year | B1-R1443X |  |
| BRCA1-30 | Unaffected | B1-R1443X |  |
| BRCA1-31 | Ovarian cancer at 49 year | B1-E352X |  |
| BRCA1-32 | Breast cancer at 52 year | B1-4160delAG |  |
| BRCA1-33 | Breast cancer at 34, 44 and 51 year | B1-R1443X |  |
| BRCA1-34 | Breast cancer at 44 year | B1-1723del9ins13 |  |
| BRCA1-35 | Unaffected | B1-2244insA |  |
| BRCA1-36 | Breast cancer at 36 year | B1-R1443X |  |
| BRCA1-37 | Unaffected | B1-2244insA |  |
| BRCA1-38 | Unaffected | B1-R1443X |  |
| BRCA1-39 | Breast cancer at 55 and 59 year | B1-2953del3+C |  |
| BRCA1-40 | Ovarian cancer at 59 year | B1-R1443X |  |
| BRCA1-41 | Breast cancer at 42 year | B1-R1443X |  |
| BRCA1-42 | Unaffected | B1-R1443X |  |
| BRCA1-43 | Placental channel cancer at 23 year | B1-2244insA |  |
| BRCA1-2 | Breast cancer at 37 and 44 year | B1-R1443X |  |
| BRCA1-3 | Breast cancer at 48 and 51 year | B1-R1443X |  |
| BRCA2-10 | Breast cancer at 44 and 49 year | B2-8765delAG | With BRCA2-17 and BRCA2-18 |
| BRCA2-11 | Breast cancer at 44 year | B2-8765delAG |  |
| BRCA2-12 | Breast cancer at 63 and ovarian cancer at 65 year | B2-8765delAG |  |
| BRCA2-13 | Breast cancer at 56 and 57 year | B2-8765delAG | With BRCA2-14 and BRCA2-15 |
| BRCA2-14 | Unaffected | B2-8765delAG | With BRCA2-13 and BRCA2-15 |
| BRCA2-15 | Unaffected | B2-8765delAG | With BRCA2-13 and BRCA2-14 |
| BRCA2-16 | Breast cancer at 76 and fallopian tube cancer at 46 year | B2-8765delAG |  |
| BRCA2-17 | Breast cancer at 29 year | B2-8765delAG | With BRCA2-10 and BRCA2-18 |
| BRCA2-18 | Breast cancer at 54 and 59 year | B2-8765delAG | With BRCA2-10 and BRCA2-17 |
| BRCA2-19 | Breast cancer at 46 year | B2-R3128X |  |
| BRCA2-20 | Uterus cancer at 37 year | B2-8765delAG |  |
| BRCA2-21 | Breast cancer at 54 year | B2-8765delAG | With BRCA2-22 |
| BRCA2-22 | Breast cancer at 50 year | B2-8765delAG | With BRCA2-21 |
| BRCA2-23 | Breast cancer at 36 year | B2-8765delAG |  |
| BRCA2-24 | Unaffected | B2-8765delAG |  |
| BRCA2-25 | Breast cancer at 28 and 60 year | B2-8765delAG | With BRCA2-26 |
| BRCA2-26 | Breast cancer at 65 year | B2-8765delAG | With BRCA2-25 |
| BRCA2-27 | Ovarian cancer at 63 year | B2-8765delAG |  |
| BRCA2-28 | Unaffected | B2-8765delAG | With BRCA2-29 |
| BRCA2-29 | Breast cancer at 36 and 63 year | B2-8765delAG | With BRCA2-28 |
| BRCA2-30 | Ovarian cancer at 50 and breast cancer at 58 year | B2-8765delAG | With BRCA2-31 |
| BRCA2-31 | Unaffected | B2-8765delAG | With BRCA2-30 |
| BRCA2-32 | Breast cancer at 41 year | B2-8765delAG | With BRCA2-33 BRCA2-34 and BRCA2-35 |
| BRCA2-33 | Lung cancer at 53 year | B2-8765delAG | With BRCA2-32 BRCA2-34 and BRCA2-35 |
| BRCA2-34 | Unaffected | B2-8765delAG | With BRCA2-32 BRCA2-33 and BRCA2-35 |
| BRCA2-35 | Unaffected | B2-8765delAG | With BRCA2-32 BRCA2-33 and BRCA2-34 |
| BRCA2-36 | Breast cancer at 49 year | B2-8765delAG | With BRCA2-37 and BRCA2-38 |
| BRCA2-37 | Unaffected | B2-8765delAG | With BRCA2-36 and BRCA2-38 |
| BRCA2-38 | Unaffected | B2-8765delAG | With BRCA2-36 and BRCA2-37 |
| BRCA2-39 | Breast cancer at 52 year | B2-8765delAG | With BRCA2-40 BRCA2-41 and BRCA2-42 |
| BRCA2-40 | Breast cancer at 57 year | B2-8765delAG | With BRCA2-39 BRCA2-41 and BRCA2-42 |
| BRCA2-41 | Unaffected | B2-8765delAG | With BRCA2-39 BRCA2-40 and BRCA2-42 |
| BRCA2-42 | Unaffected | B2-8765delAG | With BRCA2-39 BRCA2-40 and BRCA2-41 |
| BRCA2-43 | Breast cancer at 48 year | B2-8765delAG |  |
| BRCA2-44 | Unaffected | B2-8765delAG |  |
| BRCA2-45 | Breast cancer at 47 year | B2-E3002K |  |
| BRCA2-46 | Unaffected | B2-8765delAG |  |
| BRCA2-47 | Unaffected | B2-8765delAG |  |
| BRCA2-48 | Breast cancer at 50 year | B2-6503delTT |  |
| BRCA2-49 | Unaffected | B2-8765delAG |  |
| BRCA2-50 | Breast cancer at 35 and 37 year | B2-8765delAG |  |
| BRCA2-51 | Unaffected | B2-3036del4 |  |
| BRCA2-52 | Unaffected | B2-8765delAG |  |
| BRCA2-53 | Unaffected | B2-8765delAG |  |
| BRCA2-54 | Breast cancer at 41 year | B2-E3002K |  |
| BRCA2-55 | Breast cancer 48 and 60 year | B2-8765delAG |  |
| BRCA2-56 | Breast cancer at 40 year | B2-8765delAG |  |
| BRCA2-2 | Breast cancer at 47 and 53 year | B2-8765delAG |  |
| BRCA2-3 | Breast cancer at 39, 49 and 54 year | B2-8765delAG |  |
| BRCAX-1 | Breast cancer at 54 year | NA | With BRCAX-4 |
| BRCAX-3 | Breast cancer at 47 and 59 year | NA | With BRCAX-6 |
| BRCAX-11 | Breast cancer at 50 year | NA | With BRCAX-31 |
| BRCAX-12 | Breast cancer at 69 year | NA | With BRCAX-32 |
| BRCAX-13 | Breast cancer at 48 year | NA | With BRCAX-33 |
| BRCAX-14 | Breast cancer at 61 year | NA | With BRCAX-34 |
| BRCAX-15 | Breast cancer at 41 year | NA | With BRCAX-35 |
| BRCAX-16 | Breast cancer at 58 year | NA | With BRCAX-36 |
| BRCAX-17 | Breast cancer at 62 year | NA | With BRCAX-37 |
| BRCAX-18 | Breast cancer at 48 year | NA | With BRCAX-38 |
| BRCAX-19 | Breast cancer at 50 year | NA | With BRCAX-39 |
| BRCAX-20 | Breast cancer at 25 year | NA | With BRCAX-40 |
| BRCAX-21 | Breast cancer at 59 year | NA | With BRCAX-41 |
| BRCAX-22 | Breast cancer at 43 year | NA | With BRCAX-42 |
| BRCAX-23 | Breast cancer at 50 year | NA | With BRCAX-43 |
| BRCAX-24 | Breast cancer at 60 year | NA | With BRCAX-44 |
| BRCAX-4 | Unaffected | NA | With BRCAX-1 |
| BRCAX-6 | Unaffected | NA | With BRCAX-3 |
| BRCAX-31 | Unaffected | NA | With BRCAX-11 |
| BRCAX-32 | Unaffected | NA | With BRCAX-12 |
| BRCAX-33 | Unaffected | NA | With BRCAX-13 |
| BRCAX-34 | Unaffected | NA | With BRCAX-14 |
| BRCAX-35 | Unaffected | NA | With BRCAX-15 |
| BRCAX-36 | Unaffected | NA | With BRCAX-16 |
| BRCAX-37 | Unaffected | NA | With BRCAX-17 |
| BRCAX-38 | Unaffected | NA | With BRCAX-18 |
| BRCAX-39 | Unaffected | NA | With BRCAX-19 |
| BRCAX-40 | Unaffected | NA | With BRCAX-20 |
| BRCAX-41 | Unaffected | NA | With BRCAX-21 |
| BRCAX-42 | Unaffected | NA | With BRCAX-22 |
| BRCAX-43 | Unaffected | NA | With BRCAX-23 |
| BRCAX-44 | Unaffected | NA | With BRCAX-24 |

* - = No other family members included

NA= Not applicable
