# Supplementary material for: Transcriptional signature of lymphoblastoid cell lines of BRCA1, BRCA2 and non-BRCA1/2 high risk breast cancer families
Source: Oncotarget. 2017 Aug 12;8(45):78691–712. doi: 10.18632/oncotarget.20219 (PMC5667991; doi:10.18632/oncotarget.20219)
Supplement: Supplementary file 3 [file oncotarget-08-78691-s003.docx]

| **Supplementary Table 2: Anova test results** | | | | | | | | | | | | | | | | |
| --- | --- | --- | --- | --- | --- | --- | --- | --- | --- | --- | --- | --- | --- | --- | --- | --- |
| **Ensembl transcript ID** | **Gene symbol** | **Anova p value** | **Bonf Corr p value** | **Critical difference** | **Scheffe association** | | | | **Scheffe mean** | | | | **Scheffe S.D.** | | | |
|  |  |  |  |  | **G1** | **G2** | **G3** | **G4** | **G1** | **G2** | **G3** | **G4** | **G1** | **G2** | **G3** | **G4** |
| ENST00000419477 | YWHAZ | 6.7E-09 | 0.001 | 20.3 | a | a | a | b | 63.7 | 62.2 | 45.9 | 18.1 | 23.3 | 21 | 35.5 | 15.9 |
| ENST00000539269 | CARS2 | 1.2E-08 | 0.002 | 2 | a | a | a | b | 8.05 | 7.42 | 6.31 | 3.58 | 2.44 | 2.45 | 2.48 | 1.7 |
| ENST00000598296 | NOSIP | 6.7E-12 | 1E-06 | 1.93 | a | a | b | b | 4.68 | 3.91 | 0.5 | 0.28 | 2.89 | 2.36 | 0.69 | 0.73 |
| ENST00000580799 | GGA3 | 2.3E-09 | 4E-04 | 4.23 | a | a | b | b | 13.3 | 12.5 | 6.38 | 4.81 | 5.21 | 5.6 | 3.55 | 3.08 |
| ENST00000430762 | PPP3CB | 6.4E-09 | 0.001 | 1.9 | a | a | b | b | 6.29 | 6.15 | 4.26 | 2.19 | 2.39 | 2.33 | 2.15 | 1.37 |
| ENST00000486593 | LAMP2 | 6E-09 | 0.001 | 1.68 | a | a | b | b | 5.23 | 5.31 | 3.09 | 1.79 | 1.7 | 2.28 | 1.78 | 1.39 |
| ENST00000366726 | GUK1 | 6.8E-09 | 0.001 | 10 | a | a | b | b | 21.6 | 21.1 | 4.06 | 3.4 | 12.6 | 13.5 | 3.51 | 5.38 |
| ENST00000438462 | RTN4 | 9.4E-09 | 0.002 | 4.77 | a | a | b | b | 16.3 | 17.3 | 10.6 | 6.75 | 4.46 | 6.17 | 6.04 | 3.91 |
| ENST00000588730 | C18orf25 | 1.6E-08 | 0.003 | 0.78 | a | a | b | b | 1.86 | 1.91 | 0.83 | 0.34 | 0.87 | 1.04 | 0.83 | 0.45 |
| ENST00000471658 | PSPC1 | 2.4E-08 | 0.004 | 1.6 | a | a | b | b | 9.56 | 9.32 | 7.38 | 6.51 | 1.68 | 2.07 | 1.71 | 1.89 |
| ENST00000460462 | RAP2C | 7.1E-09 | 0.001 | 2.71 | ab | a | bc | c | 6.67 | 7.6 | 3.95 | 0.95 | 2.49 | 3.59 | 3.19 | 1.18 |
| ENST00000490523 | EIF2AK1 | 1.9E-12 | 3E-07 | 2.76 | b | b | a | a | 6.45 | 6.98 | 12.5 | 12.9 | 2.1 | 2.74 | 3.32 | 5.37 |
| ENST00000586868 | TBCB | 2.7E-12 | 5E-07 | 4.11 | b | b | a | a | 2.83 | 2.43 | 9.2 | 13.3 | 3.29 | 3.37 | 8.52 | 4.94 |
| ENST00000572932 | NOMO3 | 8.9E-12 | 2E-06 | 6.05 | b | b | a | a | 9.83 | 8.89 | 20.1 | 24.2 | 4.88 | 3.94 | 13.7 | 6.69 |
| ENST00000596417 | EEF2 | 6.3E-12 | 1E-06 | 104 | b | b | a | a | 91.1 | 88.5 | 366 | 299 | 67.8 | 48.7 | 232 | 122 |
| ENST00000485280 | RAB7A | 1.1E-11 | 2E-06 | 10.3 | b | b | a | a | 17.1 | 14 | 40.8 | 38.4 | 9.29 | 9.25 | 13.2 | 13.4 |
| ENST00000587393 | AES | 1.9E-11 | 3E-06 | 17.5 | b | b | a | a | 11.1 | 11.9 | 44.6 | 50.4 | 9.26 | 11.1 | 19.6 | 44.6 |
| ENST00000593582 | TRIM28 | 2.8E-11 | 5E-06 | 4.75 | b | b | a | a | 3.75 | 5.59 | 13 | 14.5 | 3.78 | 4.69 | 8.11 | 8.11 |
| ENST00000463243 | HLA-DPA1 | 6E-11 | 1E-05 | 8.82 | b | b | a | a | 14.8 | 17.2 | 30.1 | 34.8 | 6.37 | 10.6 | 12.2 | 14.5 |
| ENST00000476642 | HLA-DPA1 | 6E-11 | 1E-05 | 8.82 | b | b | a | a | 14.8 | 17.2 | 30.1 | 34.8 | 6.37 | 10.6 | 12.2 | 14.5 |
| ENST00000480481 | HLA-DPA1 | 6E-11 | 1E-05 | 8.82 | b | b | a | a | 14.8 | 17.2 | 30.1 | 34.8 | 6.37 | 10.6 | 12.2 | 14.5 |
| ENST00000483480 | HLA-DPA1 | 6E-11 | 1E-05 | 8.82 | b | b | a | a | 14.8 | 17.2 | 30.1 | 34.8 | 6.37 | 10.6 | 12.2 | 14.5 |
| ENST00000486449 | HLA-DPA1 | 6E-11 | 1E-05 | 8.82 | b | b | a | a | 14.8 | 17.2 | 30.1 | 34.8 | 6.37 | 10.6 | 12.2 | 14.5 |
| ENST00000493893 | COMT | 6.1E-11 | 1E-05 | 2.94 | b | b | a | a | 5.66 | 6.04 | 11.4 | 12 | 3.22 | 3.57 | 2.98 | 3.39 |
| ENST00000495074 | HLA-DPA1 | 6E-11 | 1E-05 | 8.82 | b | b | a | a | 14.8 | 17.2 | 30.1 | 34.8 | 6.37 | 10.6 | 12.2 | 14.5 |
| ENST00000514979 | HLA-DPA1 | 6E-11 | 1E-05 | 8.82 | b | b | a | a | 14.8 | 17.2 | 30.1 | 34.8 | 6.37 | 10.6 | 12.2 | 14.5 |
| ENST00000524786 | DEAF1 | 4.1E-11 | 7E-06 | 2.34 | b | b | a | a | 1.01 | 0.58 | 5.84 | 5.87 | 1.46 | 1.08 | 3.22 | 5.34 |
| ENST00000368439 | CKS1B | 8.1E-11 | 1E-05 | 2.78 | b | b | a | a | 7.92 | 7.91 | 13.3 | 13.8 | 3.27 | 3.18 | 3.25 | 2.37 |
| ENST00000524815 | PACS1 | 1.1E-10 | 2E-05 | 6.06 | b | b | a | a | 8.67 | 9.88 | 17.5 | 22.9 | 4.74 | 6.51 | 9.56 | 10.1 |
| ENST00000515540 | BAX | 1.9E-10 | 3E-05 | 29.4 | b | b | a | a | 61.4 | 63.6 | 118 | 122 | 22 | 27.3 | 50.5 | 48.7 |
| ENST00000548861 | RP11-603J24.9 | 2.3E-10 | 4E-05 | 11 | b | b | a | a | 14.7 | 15 | 33.2 | 38.2 | 12.5 | 12.9 | 13.9 | 9.96 |
| ENST00000529698 | DGKZ | 3.5E-10 | 6E-05 | 1.91 | b | b | a | a | 3.86 | 4.16 | 6.69 | 8.11 | 1.75 | 2.28 | 1.85 | 3.22 |
| ENST00000372077 | VEGFA | 5.3E-10 | 9E-05 | 1.96 | b | b | a | a | 0.55 | 0.84 | 2.8 | 5.14 | 1.18 | 1.64 | 3.56 | 3.85 |
| ENST00000435720 | PSMF1 | 8.7E-10 | 2E-04 | 6.29 | b | b | a | a | 18.1 | 19.5 | 28.7 | 31 | 5.98 | 5.78 | 12.3 | 8.33 |
| ENST00000461760 | STK25 | 8.9E-10 | 2E-04 | 6.14 | b | b | a | a | 18.5 | 19.1 | 30.4 | 30.3 | 7.72 | 6.69 | 5.56 | 7.5 |
| ENST00000492277 | RPL29 | 8.3E-10 | 1E-04 | 4.67 | b | b | a | a | 9.59 | 10.3 | 17.4 | 19.2 | 4.03 | 5.14 | 6.74 | 7.49 |
| ENST00000236957 | EEF1B2 | 9.7E-10 | 2E-04 | 35.1 | b | b | a | a | 81.3 | 80.3 | 124 | 160 | 24.9 | 27.4 | 79.2 | 46.6 |
| ENST00000308774 | TRMT112 | 1.2E-09 | 2E-04 | 9.14 | b | b | a | a | 21.3 | 20.9 | 37.5 | 39.2 | 8.1 | 10.7 | 9.89 | 13.6 |
| ENST00000494862 | HDLBP | 1.7E-09 | 3E-04 | 9.52 | b | b | a | a | 1.62 | 2.69 | 13.9 | 22.4 | 2.83 | 6.48 | 14.5 | 24 |
| ENST00000473991 | PSMD2 | 1.8E-09 | 3E-04 | 2.65 | b | b | a | a | 0.13 | 0.17 | 4.57 | 5.36 | 0.33 | 0.43 | 4.5 | 6.99 |
| ENST00000394729 | PRKCD | 2.3E-09 | 4E-04 | 4.75 | b | b | a | a | 16.9 | 16.9 | 24.2 | 26.5 | 4.97 | 4.38 | 8.11 | 6.43 |
| ENST00000563039 | SPN | 2.3E-09 | 4E-04 | 5.65 | b | b | a | a | 20.7 | 20.1 | 27.3 | 33 | 6.44 | 6.65 | 5.64 | 6.62 |
| ENST00000406984 | FTH1P15 | 3.9E-09 | 7E-04 | 1.48 | b | b | a | a | 1.93 | 1.8 | 4.7 | 4.6 | 1.29 | 1.77 | 1.81 | 1.88 |
| ENST00000585935 | RAVER1 | 3.9E-09 | 7E-04 | 2.48 | b | b | a | a | 2.9 | 2.93 | 7.1 | 7.61 | 2.59 | 2.42 | 3.38 | 3.91 |
| ENST00000528296 | RPL8 | 4.3E-09 | 7E-04 | 0.35 | b | b | a | a | 0.5 | 0.66 | 1.37 | 1.04 | 0.25 | 0.37 | 0.56 | 0.45 |
| ENST00000456311 | CAD | 4.5E-09 | 8E-04 | 5.83 | b | b | a | a | 12.4 | 12.9 | 20.3 | 24.4 | 6.14 | 6.27 | 8.07 | 8.49 |
| ENST00000595355 | GINS2 | 5.2E-09 | 9E-04 | 1.69 | b | b | a | a | 2.54 | 2.93 | 5.29 | 5.82 | 1.48 | 2.11 | 2.18 | 2.42 |
| ENST00000620429 | VPS11 | 5.3E-09 | 9E-04 | 0.45 | b | b | a | a | 0.16 | 0.22 | 0.66 | 1.13 | 0.34 | 0.38 | 0.95 | 0.7 |
| ENST00000630977 | VPS11 | 5.3E-09 | 9E-04 | 0.45 | b | b | a | a | 0.16 | 0.22 | 0.66 | 1.13 | 0.34 | 0.38 | 0.95 | 0.7 |
| ENST00000352980 | KAT5 | 5.7E-09 | 1E-03 | 0.37 | b | b | a | a | 0.45 | 0.55 | 1.1 | 1.14 | 0.28 | 0.35 | 0.55 | 0.72 |
| ENST00000456818 | TUBA4A | 6.4E-09 | 0.001 | 8.03 | b | b | a | a | 8.88 | 8.71 | 20.6 | 24.6 | 6.52 | 5.37 | 14.8 | 15.4 |
| ENST00000517577 | FTH1P11 | 6.4E-09 | 0.001 | 0.28 | b | b | a | a | 0.44 | 0.48 | 0.96 | 0.94 | 0.25 | 0.36 | 0.3 | 0.38 |
| ENST00000591301 | GNA11 | 6.2E-09 | 0.001 | 0.68 | b | b | a | a | 0.58 | 0.83 | 1.82 | 1.85 | 0.51 | 0.65 | 1.05 | 1.36 |
| ENST00000523037 | MRPL22 | 6.7E-09 | 0.001 | 0.14 | b | b | a | a | 0.5 | 0.51 | 0.71 | 0.76 | 0.15 | 0.17 | 0.19 | 0.13 |
| ENST00000606722 | NDUFA13 | 7.6E-09 | 0.001 | 15 | b | b | a | a | 20.2 | 22.3 | 39.7 | 50.8 | 11.5 | 14.5 | 30.6 | 20.7 |
| ENST00000381348 | LINC00634 | 8.2E-09 | 0.001 | 0.3 | b | b | a | a | 0.53 | 0.61 | 0.92 | 1.16 | 0.31 | 0.28 | 0.32 | 0.64 |
| ENST00000594493 | RPS11 | 1.1E-08 | 0.002 | 1.19 | b | b | a | a | 3.99 | 4.01 | 6.41 | 5.96 | 1.21 | 1 | 2.03 | 1.61 |
| ENST00000568265 | TAF1C | 1.2E-08 | 0.002 | 1.36 | b | b | a | a | 0.63 | 0.9 | 3.91 | 2.66 | 0.71 | 1 | 3.08 | 1.61 |
| ENST00000597681 | MAP1S | 1.3E-08 | 0.002 | 4.74 | b | b | a | a | 5.65 | 5.56 | 12.7 | 14.5 | 4.21 | 4.17 | 6.39 | 9.36 |
| ENST00000368436 | CKS1B | 1.6E-08 | 0.003 | 1.39 | b | b | a | a | 2.63 | 2.62 | 4.98 | 5.1 | 1.7 | 1.6 | 1.87 | 0.89 |
| ENST00000537533 | PTPN6 | 2.4E-08 | 0.004 | 26.6 | b | b | a | a | 11.4 | 12.5 | 41.9 | 64.6 | 17 | 23.8 | 47.2 | 51 |
| ENST00000569760 | FUS | 2.4E-08 | 0.004 | 1.59 | b | b | a | a | 1.44 | 1.47 | 3.21 | 4.63 | 1.05 | 1.52 | 3.24 | 2.32 |
| ENST00000533397 | RPL8 | 2.5E-08 | 0.004 | 2.31 | b | b | a | a | 6.61 | 6.98 | 11.4 | 10.3 | 1.96 | 2.43 | 3.21 | 3.6 |
| ENST00000443451 | NCOR2 | 3.2E-08 | 0.006 | 3.89 | b | b | a | a | 10.1 | 10.5 | 16.7 | 16.9 | 3.86 | 4.1 | 4.85 | 6.39 |
| ENST00000487513 | EHMT2 | 3.9E-08 | 0.007 | 1.53 | b | b | a | a | 1.07 | 1.18 | 3.3 | 3.83 | 1.55 | 1.39 | 2.29 | 2.66 |
| ENST00000552600 | ESPL1 | 4.9E-08 | 0.008 | 2.42 | b | b | a | a | 4.14 | 4.36 | 8.05 | 8.3 | 2.74 | 2.74 | 3.27 | 2.73 |
| ENST00000543608 | SPPL3 | 5.7E-08 | 0.01 | 3.16 | b | b | a | a | 12.2 | 13 | 18.3 | 17.2 | 3.43 | 3.04 | 3.94 | 5.25 |
| ENST00000436614 | ZNF687 | 3.3E-12 | 6E-07 | 0.95 | b | b | b | a | 0.45 | 0.58 | 1.28 | 3.2 | 0.4 | 0.73 | 1.4 | 2.13 |
| ENST00000237837 | FGF23 | 4E-11 | 7E-06 | 3.39 | b | b | b | a | 1.31 | 1.11 | 4.21 | 10.4 | 1.32 | 0.94 | 3.82 | 9.25 |
| ENST00000452722 | CADM1 | 3.5E-10 | 6E-05 | 0.11 | b | b | b | a | 0.08 | 0.07 | 0.16 | 0.37 | 0.05 | 0.05 | 0.14 | 0.3 |
| ENST00000459748 | RP11-466H18.1 | 9.1E-10 | 2E-04 | 62 | b | b | b | a | 3.04 | 3.15 | 40.9 | 160 | 13.3 | 22 | 84.2 | 165 |
| ENST00000460469 | NMD3 | 8.9E-10 | 2E-04 | 0.89 | b | b | b | a | 0.99 | 1.06 | 1.52 | 3.27 | 0.5 | 1.04 | 0.96 | 1.64 |
| ENST00000562465 | CDAN1 | 8.5E-10 | 1E-04 | 0.34 | b | b | b | a | 0.82 | 0.93 | 1.14 | 1.64 | 0.33 | 0.44 | 0.29 | 0.47 |
| ENST00000495645 | CHPF2 | 2.4E-09 | 4E-04 | 0.58 | b | b | b | a | 0.15 | 0.21 | 0.58 | 1.54 | 0.22 | 0.54 | 0.66 | 1.37 |
| ENST00000377861 | PCDH9 | 3.6E-09 | 6E-04 | 0.05 | b | b | b | a | 0.08 | 0.08 | 0.11 | 0.2 | 0.03 | 0.03 | 0.07 | 0.11 |
| ENST00000415265 | WDR6 | 3.8E-09 | 7E-04 | 5.77 | b | b | b | a | 5.51 | 5.71 | 10.6 | 18.7 | 5.83 | 6.1 | 7.91 | 8.53 |
| ENST00000552588 | RPL18 | 4.9E-09 | 9E-04 | 0.68 | b | b | b | a | 1.11 | 1.14 | 1.46 | 2.76 | 0.67 | 0.72 | 0.74 | 1.09 |
| ENST00000374752 | ACAD8 | 8.3E-09 | 0.001 | 0.41 | b | b | b | a | 0.04 | 0.14 | 0.43 | 0.95 | 0.15 | 0.26 | 0.61 | 1.06 |
| ENST00000449683 | ATP5J2 | 1E-08 | 0.002 | 13.4 | b | b | b | a | 19 | 18.4 | 29.2 | 49 | 12.3 | 12.7 | 24.2 | 17.7 |
| ENST00000513391 | OCIAD1 | 1.1E-08 | 0.002 | 1.12 | b | b | b | a | 0.73 | 0.88 | 1.65 | 3.22 | 0.94 | 0.97 | 1.83 | 2.09 |
| ENST00000547276 | HNRNPA1 | 1.4E-08 | 0.002 | 19.8 | b | b | b | a | 24.4 | 21.9 | 40.7 | 67.5 | 17 | 14.1 | 37 | 33.9 |
| ENST00000525085 | NDUFC2 | 1.8E-08 | 0.003 | 8.12 | b | b | b | a | 30.1 | 31.9 | 36.7 | 48.1 | 7.13 | 10.6 | 9.96 | 10.6 |
| ENST00000500813 | DCTD | 1.9E-08 | 0.003 | 0.49 | b | b | b | a | 1.2 | 1.2 | 1.61 | 2.27 | 0.42 | 0.44 | 0.96 | 0.72 |
| ENST00000612832 | ARHGAP21 | 2.4E-08 | 0.004 | 0.15 | b | b | b | a | 0.01 | 0.04 | 0.11 | 0.34 | 0.03 | 0.11 | 0.24 | 0.36 |
| ENST00000535413 | MLEC | 2.8E-08 | 0.005 | 4.71 | b | b | b | a | 6.16 | 6.64 | 9.95 | 16.3 | 4.22 | 4.38 | 7.21 | 8.53 |
| ENST00000498022 | NAGK | 2.9E-08 | 0.005 | 1.57 | b | b | b | a | 0.48 | 0.6 | 1.78 | 3.83 | 0.69 | 0.84 | 2.13 | 4.16 |
| ENST00000444034 | MED12 | 3.2E-08 | 0.005 | 1.11 | b | b | b | a | 1.69 | 2.03 | 2.53 | 4.12 | 0.78 | 1.25 | 1.27 | 2.19 |
| ENST00000522754 | NCALD | 3.5E-08 | 0.006 | 0.81 | b | b | b | a | 0.87 | 0.78 | 1.28 | 2.7 | 0.44 | 0.39 | 0.94 | 2.11 |
| ENST00000552819 | PCBP2 | 1.3E-08 | 0.002 | 16.8 | bc | c | b | a | 65.7 | 63.1 | 80.9 | 102 | 17.6 | 17.9 | 26.9 | 16.6 |
| ENST00000528413 | IRF7 | 1.6E-08 | 0.003 | 4.79 | bc | c | b | a | 3.51 | 1.95 | 6.7 | 14.1 | 4.79 | 2.83 | 6.68 | 9.14 |
| ENST00000466397 | RPL29 | 2.4E-08 | 0.004 | 0.21 | c | bc | a | ab | 0.24 | 0.36 | 0.74 | 0.56 | 0.17 | 0.22 | 0.3 | 0.32 |
| ENST00000586270 | H3F3B | 1.1E-08 | 0.002 | 36.4 | c | bc | ab | a | 39.9 | 50.6 | 83.1 | 116 | 33.6 | 39.8 | 66.7 | 45 |
| ENST00000405878 | XRCC6 | 6.4E-13 | 1E-07 | 19.2 | c | c | b | a | 15.3 | 19.6 | 40.7 | 69.8 | 12.1 | 22.5 | 32.4 | 26.8 |
| ENST00000427834 | SGSM3 | 8E-12 | 1E-06 | 0.85 | c | c | b | a | 0.52 | 0.54 | 1.55 | 2.77 | 0.6 | 0.66 | 1.45 | 1.62 |
| ENST00000537739 | HDGF | 2.6E-11 | 5E-06 | 5.94 | c | c | b | a | 3.88 | 3.66 | 11 | 19 | 4.41 | 6.5 | 10.1 | 7.69 |

Bonf Corr p value - Anova p-value after Bonferroni correcton; Critical difference- Critical value to reject the null hypothesis with the Scheffe test; G1-BRCA1; G2-BRCA2; G3- BRCAX unaffected; G4- BRCAX affected; Scheffe association- Association of each BRCA sample group with the group defined by the Scheffe test (i.e.: two BRCA groups will be in the same Scheffe group if the difference between their mean TPM values is smaller than the critical difference); Scheffe Mean- Mean TPM values of each group used for the Scheffe test; Scheffe S.D.- Standard deviation of the TPM values for each group used for the Scheffe test
