# Supplementary material for: Transcriptional signature of lymphoblastoid cell lines of BRCA1, BRCA2 and non-BRCA1/2 high risk breast cancer families
Source: Oncotarget. 2017 Aug 12;8(45):78691–712. doi: 10.18632/oncotarget.20219 (PMC5667991; doi:10.18632/oncotarget.20219)
Supplement: Supplementary file 4 [file oncotarget-08-78691-s004.docx]

| **Supplementary Table 3: Transcripts associated with each subgroup** | | | |
| --- | --- | --- | --- |
| **Ensembl transcript** | **Gene symbol** | **Ensembl transcript** | **Gene Symbol** |
| ***BRCA1*** | | ***BRCA1* and *BRCA2* (continued)** | |
| ENST00000586270 | *H3F3B* | ENST00000587393 | *AES* |
|  |  | ENST00000593582 | *TRIM28* |
| ***BRCA2*** | | ENST00000463243 | *HLA-DPA1* |
| ENST00000460462 | *RAP2C* | ENST00000476642 | *HLA-DPA1* |
|  |  | ENST00000480481 | *HLA-DPA1* |
| ***BRCA2* and BRCAX affected** | | ENST00000483480 | *HLA-DPA1* |
| ENST00000552819 | *PCBP2* | ENST00000486449 | *HLA-DPA1* |
| ENST00000528413 | *IRF7* | ENST00000493893 | *COMT* |
|  |  | ENST00000495074 | *HLA-DPA1* |
| ***BRCA1, BRCA2* and BRCAX affected** | | ENST00000514979 | *HLA-DPA1* |
| ENST00000405878 | *XRCC6* | ENST00000524786 | *DEAF1* |
| ENST00000427834 | *SGSM3* | ENST00000368439 | *CKS1B* |
| ENST00000537739 | *HDFG* | ENST00000524815 | *PACS1* |
|  |  | ENST00000515540 | *BAX* |
| **BRCAX affected** | | ENST00000548861 | *RP11-603J24.9* |
| ENST00000419477 | *YWHAZ* | ENST00000529698 | *DGKZ* |
| ENST00000539269 | *CARS2* | ENST00000372077 | *VEGFA* |
| ENST00000436614 | *ZNF687* | ENST00000435720 | *PSMF1* |
| ENST00000237837 | *FGF23* | ENST00000461760 | *STK25* |
| ENST00000452722 | *CADM1* | ENST00000492277 | *RPL29* |
| ENST00000459748 | *RP11-466H18.1* | ENST00000236957 | *EEF1B2* |
| ENST00000460469 | *NMD3* | ENST00000308774 | *TRMT112* |
| ENST00000562465 | *CDAN1* | ENST00000494862 | *HDLBP* |
| ENST00000495645 | *CHPF2* | ENST00000473991 | *PSMD2* |
| ENST00000377861 | *PCDH9* | ENST00000394729 | *PRKCD* |
| ENST00000415265 | *WDR6* | ENST00000563039 | *SPN* |
| ENST00000552588 | *RPL18* | ENST00000406984 | *FTH1P15* |
| ENST00000374752 | *ACAD8* | ENST00000585935 | *RAVER1* |
| ENST00000449683 | *ATP5J2* | ENST00000528296 | *RPL8* |
| ENST00000513391 | *OCIAD1* | ENST00000456311 | *CAD* |
| ENST00000547276 | *HNRNPA1* | ENST00000595355 | *GINS2* |
| ENST00000525085 | *NDUFC2* | ENST00000620429 | *VPS11* |
| ENST00000500813 | *DCTD* | ENST00000630977 | *VPS11* |
| ENST00000612832 | *ARHGAP21* | ENST00000352980 | *KAT5* |
| ENST00000535413 | *MLEC* | ENST00000456818 | *TUBA4A* |
| ENST00000498022 | *NAGK* | ENST00000517577 | *FTH1P11* |
| ENST00000444034 | *MED12* | ENST00000591301 | *GNA11* |
| ENST00000522754 | *NCALD* | ENST00000523037 | *MRPL22* |
|  |  | ENST00000606722 | *NDUFA13* |
| ***BRCA1* and *BRCA2*** | | ENST00000381348 | *LINC00634* |
| ENST00000598296 | *NOSIP* | ENST00000594493 | *RPS11* |
| ENST00000580799 | *GGA3* | ENST00000568265 | *TAF1C* |
| ENST00000430762 | *PPP3CB* | ENST00000597681 | *MAP1S* |
| ENST00000486593 | *LAMP2* | ENST00000368436 | *CKS1B* |
| ENST00000366726 | *GUK1* | ENST00000537533 | *PTPN6* |
| ENST00000438462 | *RTN4* | ENST00000569760 | *FUS* |
| ENST00000588730 | *C18orf25* | ENST00000533397 | *RPL8* |
| ENST00000471658 | *PSPC1* | ENST00000443451 | *NCOR2* |
| ENST00000490523 | *EIF2AK1* | ENST00000487513 | *EHMT2* |
| ENST00000586868 | *TBCB* | ENST00000552600 | *ESPL1* |
| ENST00000572932 | *NOMO3* | ENST00000543608 | *SPPL3* |
| ENST00000596417 | *EEF2* | ENST00000466397 | *RPL29* |
| ENST00000485280 | *RAB7A* |  |  |
